# Mechanical and Sound Absorption Properties of Ice-Templated Porous Cement Co-Incorporated with Silica Fume and Fly Ash

**DOI:** 10.3390/ma19010092

**Published:** 2025-12-26

**Authors:** Xiaoyang Zhang, Kang Peng, Bin Xiao, Jianxin Yang, Bao Yang, Boyuan Li

**Affiliations:** 1 School of Mathematics and Physics, University of South China, Hengyang 421001, China; xyzhang@usc.edu.cn (X.Z.); 17749835893@163.com (B.L.); 2 Hunan Provincial Key Laboratory of High-Performance Special Concrete, School of Civil Engineering, University of South China, Hengyang 421001, China; 20232009110583@stu.usc.edu.cn (K.P.); xiaobin040213@163.com (B.X.); 20220650308@stu.usc.edu.cn (J.Y.); 3 School of Civil Engineering and Transportation, South China University of Technology, Guangzhou 510640, China

**Keywords:** ice-templating, cement-based material, fly ash, silica fume, mechanical properties, sound absorption performance

## Abstract

Reducing the consumption of energy-intensive cement and promoting the resource utilization of industrial waste are two critical challenges that should be urgently addressed to achieve the goals of carbon neutrality and green sustainable development in the building materials field. Among these, the massive stockpiling of industrial waste such as fly ash and silica fume poses serious threats to the environment and human health, making their efficient utilization an urgent need to alleviate environmental pressure. This study employs the ice-template method to incorporate fly ash and silica fume as functional components into a cement-based system, fabricating a novel composite material. This material features a layered porous structure, which not only reduces cement usage but also results in a lighter weight. The introduction of the ice-templating method successfully constructed an anisotropic lamellar structure, leading to significant enhancements in flexural strength and toughness—by approximately 26.6% and 30%, respectively, vertical to the lamellae compared to conventional dense cement. Meanwhile, the hybrid blend of silica fume and fly ash effectively improved the deformability of the material, as evidenced by a notable increase in compressive failure strain. These excellent behaviors of mechanical properties are attributed to the formation of a multi-scale microstructure characterized by “macroscopically continuous and microscopically dense” features. Moreover, this specific microstructure offers greater advantages in sound absorption performance. The acoustic impedance tube tests demonstrate that the noise reduction coefficient of the novel cement-based material incorporating fly ash and silica fume is improved by 82%, holding promising applications in noise reduction for the construction and transportation fields. This research provides a feasible pathway for the high-value application of industrial solid waste in low-carbon materials.

## 1. Introduction

Cement, as the most widely used construction material globally, imposes substantial environmental costs, with its production process contributing to approximately 8% of anthropogenic CO_2_ emissions [1,2]. Driven by ongoing urbanization, global cement demand will be increased by 12–23% by 2050 [3], a trend that will inevitably exacerbate carbon emission challenges. Therefore, achieving effective carbon reduction now constitutes a pivotal scientific challenge that is imperative to address. The utilization of industrial waste such as fly ash (FA) and silica fume (SF) as supplementary cementitious materials to partially replace clinker is regarded as a highly promising pathway for emission reduction [4]. However, conventional cement-based materials still commonly suffer from limitations such as relatively low strength, significant brittleness, and limited functionality, which restrict their further development. Therefore, it is significant to systematically investigate the synergistic effects of novel fabrication techniques and multi-component admixtures on the mechanical and sound absorption properties of cementitious materials for enhancing overall performance and simultaneously realizing environmental and economic benefits.

The incorporation of supplementary materials significantly influences the microstructure and mechanical properties of cementitious materials. SF, a by-product from ferrosilicon alloy smelting, consists primarily of SiO_2_ and exists as amorphous spherical particles with diameters ranging from 0.1 to 1.0 µm, exhibiting high pozzolanic reactivity [5]. Studies have shown that incorporating SF effectively optimizes the microstructure of cement-based materials, enhancing their compactness and mechanical strength [6,7]. Another supplementary material, FA, an aluminosilicate solid waste discharged from coal-fired power plants [8], is mainly composed of glassy microspheres measuring 0.5–300 µm. The usage of FA not only facilitates waste valorization and reduces carbon but also improves the workability of fresh concrete, mitigates early-age hydration heat and the risk of thermal cracking, and significantly enhances long-term mechanical performance and durability [9]. Furthermore, the unique physical and chemical characteristics of FA make it an ideal platform for developing multifunctional materials [10]. For instance, its hollow microspheres can be utilized in lightweight thermal insulation composites [11], it can be converted into zeolite molecular sieves via alkaline fusion for heavy metal adsorption [12], and it can regulate internal moisture transport to influence durability in concrete [13]. Notably, SF and FA demonstrate a significant synergistic effect when used in combination. Research by Jiang et al. [14] indicated that cement mortar incorporating 8% SF and 10% mechanically activated FA achieved over 30% higher 28-day compressive and flexural strength compared to the control group, attributed to their favorable particle packing and combined pozzolanic reactions. From a hydration mechanism perspective, Zhu et al. [15] used hydration heat analysis and demonstrated that the combined system can modulate the hydration process, reduce the exothermic peak, and positively influence later-age strength development. Furthermore, the study by Lin et al. [16] on the fiber-matrix interaction in engineered cementitious composites (ECC) also further reveals the potential of improving the macroscopic mechanical behavior of materials through microstructural design. Although using supplementary cementitious materials in combination can, to some extent, optimize the performance and environmental benefits of cement-based materials, their microstructure generally remains disordered and isotropic [17]. This inherent characteristic makes it challenging to achieve the synergistic design and precise regulation of both mechanical properties and functional characteristics in specific direction. Moreover, effects of SF and FA on mechanical and sound absorption properties of cementitious materials fabricated by advanced methods have not been explored.

The ice-templating method, as a bioinspired structural fabrication strategy, utilizes the directional growth of ice crystals during controlled freezing to expel cementitious particles into the inter-crystal spaces, thereby forming highly ordered porous lamellar architectures. These structures are subsequently preserved either by ice sublimation or through in-situ hydration fixation [18,19]. This technique enables a transition from a “disordered” to an “ordered” microstructure in materials, which not only significantly enhances mechanical performance but also imparts excellent multifunctionality, including thermal insulation, energy storage, and sound absorption. The ice-templating method demonstrates considerable advantages and potential in the field of cement-based materials. This precise control of the material’s microstructure aligns with the approach demonstrated by Xi et al. [20] in porous structure design and performance optimization. Abdullayev et al. [21] successfully fabricated low-cost cement membrane supports with high pore connectivity using a slow freeze-casting technique. Dong et al. [22,23] developed a one-step ice-casting-hydration method that enabled the controlled construction of hierarchical pore structures and effectively optimized pore morphology and material properties by adjusting solvent composition. For achieving breakthroughs in mechanical performance, the ice-templating method has effectively addressed the longstanding challenge of balancing strength and toughness in cement-based materials by constructing brick-and-mortar-like biomimetic structures. Chen et al. [24,25] created a cement-hydrogel multi-layered composite through ice-templating method, achieving a 175-fold increase in toughness while incorporating self-healing capabilities and low thermal conductivity. Wang et al. [26] developed “wood-inspired cement” that demonstrated superior strength at equal density compared to traditional foam cement, along with tunable thermal and permeable properties. The ice-templating method holds great promise in the field of advanced multifunctional materials [27,28,29,30,31,32,33,34,35]. Currently, ice-templating method has been preliminarily employed to realize the integration of structure and function in cement-based materials [24,25,26,35]. Xing et al. [35] employed bidirectional ice-templating method to prepare cement materials with aligned lamellar structures that simultaneously demonstrated excellent mechanical properties, remarkable ionic conductivity, and thermal insulation. However, there is a lack of in-depth research combining ice-templating method with composite admixtures incorporating industrial waste to synergistically regulate both the structure and functionality of cement-based materials.

The development of high-sound-absorption cement-based materials presents a promising strategy for mitigating escalating noise pollution. Scholars have conducted extensive exploration in this field. For instance, Huan et al. [36] regulated the pore structure by introducing lightweight aggregates such as ceramsite and perlite, along with fibers and air-entraining agents, to fabricate porous materials with an average sound absorption coefficient reaching 0.6. Geng Limin et al. [37] utilized FA ceramsite to produce lightweight concrete sound barriers with a Noise Reduction Coefficient (NRC) of 0.74–0.89. Recently, Du et al. [38], inspired by nature, fabricated bioinspired cement aerogels via directional freezing technology, demonstrating a high sound absorption coefficient of up to 0.8, which reveals the significant potential of aligned pore structures in enhancing acoustic functionality. Ice-templating method has demonstrated its universal advantage in preparing high-performance acoustic materials with oriented porous structures. Tian et al. [39] constructed a heterogeneous layered structure of aramid nanofiber aerogels using this method, achieving an extremely high low-frequency sound absorption coefficient of 0.99 at 880 Hz. Similarly, Li et al. [40] and Pornea et al. [41] successfully prepared poly(silsesquioxane) aerogel fibers and dual-pore network polymer aerogels, respectively, by ice-templating method. These materials exhibit favorable broadband sound absorption performance. Precise control over the pore structure is effective to break through the bottleneck of low-frequency sound absorption for materials. However, a critical scientific challenge remains unaddressed: the synergistic effects and underlying mechanisms arising from the combination of a macro-structural design strategy (ice-templating) with a micro-component optimization approach (SF/FA hybrid blend) within a single cementitious system. While ice-templating method contributes to creating ordered macro-porous structures, and SF/FA are known to densify the micro-scale matrix, their interaction is not merely additive but potentially transformative. It is hypothesized that the ice-templated lamellar skeleton can provide a continuous pathway for functional performance (e.g., sound absorption), while the SF/FA blend concurrently modifies the mechanical properties and introduces micro-porosity within the lamellae. This synergy may construct a unique hierarchical structure that overcomes the traditional performance trade-offs between mechanical strength and acoustic absorption efficiency. The specific scientific objective of this study is to disentangle and elucidate the synergy between the ice-templating process and the SF/FA admixtures—specifically, how their interaction jointly modulates the multi-scale structure, thereby governing the final mechanical and acoustic properties.

In this study, a novel cement-based composite incorporating 10% SF and 10% FA was fabricated by the ice-templating method. Uniaxial compression, three-point bending, and impedance tube tests were conducted to investigate the compressive strength, flexural performance, and sound absorption coefficient of the materials. To investigate the interplay between micro-optimization and ice-templated macro-structure, a blend of 10% SF and 10% FA (by binder mass) was adopted. This ratio leverages SF’s efficacy in ice-templated systems [26] and the proven synergistic enhancement of this hybrid blend on densification and properties [42]. The microstructure was characterized by scanning electron microscopy (SEM) to elucidate the synergistic mechanisms between the ice-templated aligned porous structure and the composite admixtures. This research offers new insights for the design and development of high-performance, multifunctional integrated cement-based composites.

## 2. Materials and Methods

### 2.1. Raw Materials

The cement used in the experiments was P.O 42.5 Portland cement, sourced from Chejiang Southern Cement Co., Ltd. (Hengyang, China). SF was produced by Wufu Environmental Technology Co., Ltd. (Zhengzhou, China). Grade I FA was supplied by the Shijiazhuang Shang’an Power Plant (Shijiazhuang, China). Their main chemical compositions are shown in Table 1. The viscosity modifier was Hydroxypropyl Methyl Cellulose (HPMC) type II, manufactured by Shanghai Aladdin Biochemical Technology Co., Ltd. (Shanghai, China) with a viscosity within the typical range for such applications. Polydimethylsiloxane (PDMS) was purchased from Dow Corning.

### 2.2. Preparation of Ice-Templated Cement-Based Composites with Silica Fume/Fly Ash

**Figure 1:**
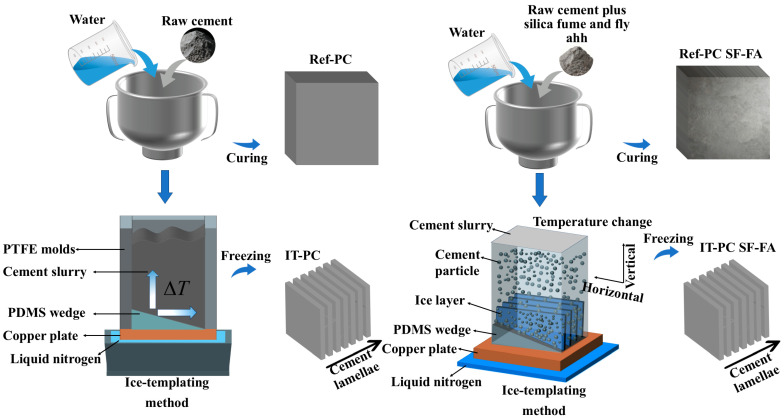
Schematic illustrations of the fabrication process.

A simplified ice-templating method was employed to fabricate the layered cement paste skeleton, with a schematic diagram of the experimental process shown in Figure 1. First, ordinary Portland cement, SF, and FA were dry-mixed and stirred at low speed for 60 s until homogenous. Subsequently, deionized water and a pre-mixed Hydroxypropyl Methyl Cellulose (HPMC) solution were added at a water-to-binder ratio of 0.8. The HPMC solution was prepared at a concentration of 1% by mass of the estimated water-to-binder ratio. HPMC powder was dissolved in deionized water under stirring prior to the mixing process to ensure its uniform dispersion in the cement paste, thereby modifying the rheology and mitigating bleeding in the high water-to-binder ratio (0.8) system, which is crucial for achieving uniform freezing. The mixture was stirred slowly for 90 s, followed by a 15 s pause to scrape off any cement residue adhering to the mixer walls, and then stirred at medium speed for another 90 s to obtain a uniform cement paste. The mixed cement paste was poured in stages into a 50 mm × 50 mm × 50 mm square polytetrafluoroethylene mold: an initial portion was placed in the center of the mold and vibrated for 60 s on a vibrating table; the mold was then filled completely with the remaining paste and vibrated for an additional 60 s until no significant air bubbles escaped.

**Figure 2:**
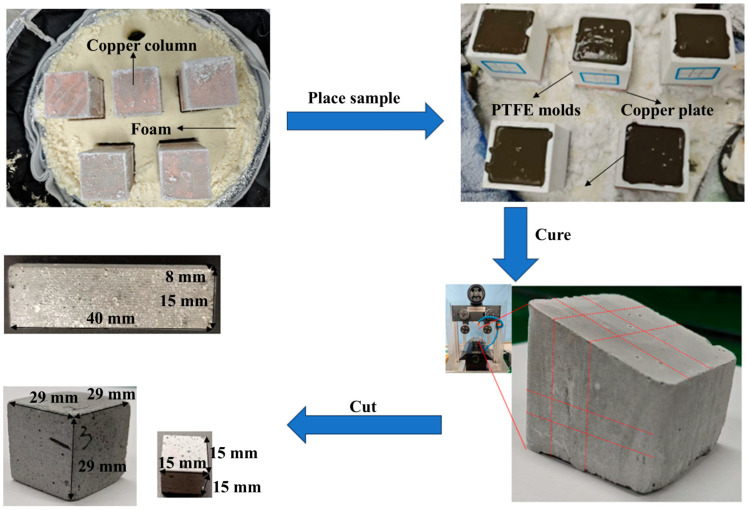
Ice-templating samples and samples for three-point bending and compression tests and sound absorption tests.

The mold was subsequently placed on top of a copper column for freezing. The bottom of the copper column was immersed in liquid nitrogen, enabling indirect heat transfer through the copper plate and column to gradually freeze the water in the paste into ice over a total freezing duration of approximately 1.5 h. To establish bidirectional temperature gradients both vertically and horizontally, a polydimethylsiloxane (PDMS) wedge with a 15° angle was pre-positioned at the bottom of the mold. The PDMS, characterized by its low thermal conductivity, created asymmetric heat transfer conditions. This setup promoted a horizontal thermal gradient from the thinner to the thicker region of the wedge, simultaneously with the vertical gradient established by the copper column, collectively guiding the directional growth of ice crystals. After complete freezing, the sample was transferred to a 2–5 °C environment and stored for 2 days to allow slow thawing. This process promotes hydration reactions between the cement particles and the melted ice, thereby stabilizing the lamellar structure formed during freezing. Finally, the thawed samples were immersed in water for curing under controlled conditions of 20 ± 2 °C and relative humidity > 95%. The cured samples are shown in Figure 2.

Four groups of samples were designed: conventionally cast plain cement paste (Ref-PC), conventionally cast cement composite with SF/FA (Ref-PC SF-FA), ice-templated plain cement paste (IT-PC), and ice-templated bio-inspired layered cement composite with SF/FA (IT-PC SF-FA).

### 2.3. Microstructural Characterization

#### 2.3.1. Morphological Analysis

Morphological analysis of cement-based composites was conducted by Scanning Electron Microscopy (SEM). The microstructure was characterized by the FEI Apreo 2S field-emission scanning electron microscope (FE-SEM) (Thermo Fisher Scientific (China) Co., Ltd., Shanghai, China). Prior to imaging, the sample surfaces were sputter-coated with a thin gold layer to enhance electrical conductivity. Observations were conducted at an accelerating voltage of 3 kV utilizing the secondary electron imaging mode to examine the surface morphology and layered structure. The magnification was adjusted as required, within a range of 65× to 1200×, to capture relevant microstructural features.

#### 2.3.2. Pore Structure Characterization

The Mercury Intrusion Porosimetry (MIP) test was conducted to characterize the pore structure. The pore structure parameters, including pore size distribution, total pore volume, specific surface area, and porosity, were quantitatively characterized using a Mercury Intrusion Porosimeter (AutoPore V 9600, Micromeritics Instrument Corporation, Norcross, GA, USA). The measurements were conducted over a wide pressure range from 0.48 psia to 61,000 psia, corresponding to a detectable pore diameter range of approximately 370,000 nm to 5 nm, based on the Washburn equation. A mercury contact angle of 130° and a surface tension of 0.485 N/m were used for the calculations. Prior to testing, the samples were vacuum-dried at 40 °C to remove moisture. The obtained intrusion and extrusion data were used to derive key parameters such as the median pore diameter, threshold pressure, and permeability, providing comprehensive insight into the pore network’s size, volume, and connectivity.

### 2.4. Mechanical Performance Testing Methods

All mechanical tests were conducted at room temperature. Test samples were prepared from the cement samples using the STX-202A precision diamond wire saw (Shenyang Kejing Auto-instrument Co., Ltd., Shenyang, China), cut to dimensions of 40 mm × 15 mm × 8 mm (for three-point bending tests) and 15 mm × 15 mm × 15 mm (for uniaxial compression tests), as illustrated in Figure 2. To ensure structural consistency, the uneven surfaces at the top and bottom of the ice-templated samples, resulting from the fabrication process, were removed by cutting; only the structurally uniform middle section was retained. Conventionally cast cement materials were cut into samples of identical dimensions for comparison. At least three replicate samples were prepared for each processing condition for mechanical testing, and the results are reported as the mean ± standard deviation.

Mechanical tests were conducted using a computer-controlled electronic universal testing machine (Sinotest Equipment Co., Ltd., Changchun, China). The three-point bending tests employed a 500 N load cell with a constant speed of 0.5 $\mathrm{mm}\cdot \min^{-1}$, while the compression tests utilized a 5 kN load cell with a constant speed of 0.15 $\mathrm{mm}\cdot \min^{-1}$, ensuring quasi-static loading conditions throughout the experiments. To investigate the anisotropic characteristics of the materials, samples were cut and loads were applied along directions vertical and parallel to the ice-templated lamellar structure, respectively. The compressive strength $f_{c}$ [33] and flexural strength $\sigma_{f}$ [28] were calculated using the following equations:

(1) $$f_{c}=\frac{F_{\max}}{A}$$

where $F_{\max}$ and A represent the maximum load recorded prior to sample failure and the compressive area, respectively.

(2) $$\sigma_{f}=\frac{3FL}{2bd^{2}}$$

where F is the applied force; L is the span length (30 mm); b is the width of the sample (15 mm); and d is the thickness of the sample (8 mm).

### 2.5. Acoustic Performance Testing Methods

The acoustic properties in this study were measured using a DY3000-R-A29 rectangular impedance tube (Suzhou Dongyuan Electronics Co., Ltd., Suzhou, China) based on the transfer function method. This system accurately determines key acoustic parameters within specific frequency ranges, including the sound absorption coefficient, reflection coefficient, surface acoustic impedance, transmission coefficient, and transmission loss. All testing was performed under ambient laboratory conditions.

For sample preparation, cement-based samples were cut into 29 mm × 29 mm × 29 mm cubes (Figure 2) using an STX-202A precision diamond wire saw (Shenyang Kejing Auto-instrument Co., Ltd., Shenyang, China). For each processing condition, at least three replicate samples were prepared for acoustic testing, and the results are reported as the mean ± standard deviation.

#### 2.5.1. Testing Principle and Equipment

**Figure 3:**
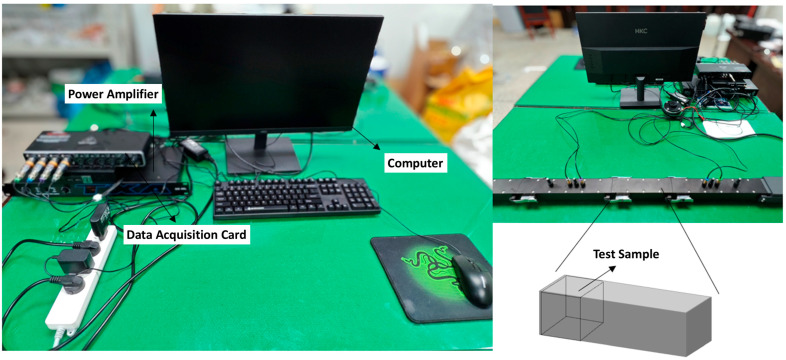
Photograph of the impedance tube testing system.

The traditional standing wave method for measuring the sound absorption coefficient involves cumbersome operation and low efficiency due to point-by-point frequency measurement. In contrast, the transfer function method adopted in this study is more efficient, as it acquires the acoustic characteristics across the entire frequency range through a single broadband signal excitation. The testing system comprises an impedance tube, microphones, a power amplifier, a data acquisition card, and computer control software. A photograph of the system setup is provided in Figure 3.

The sound absorption test was performed using the two-microphone transfer function method, complying with international standards ISO 10534 [43] and ASTM E1050 [44], as well as the Chinese national standard GB/T 18696 [45]. During testing, the sample was placed at one end of the impedance tube. A logarithmic chirp signal was generated by the loudspeaker, and the sound pressure signals were captured by microphones at two fixed positions. The complex transfer function $H_{12}=p_{2}/p_{1}$ was calculated, from which the complex reflection coefficient r was derived as follows:

(3) $$r=\frac{H_{12}-H_{l}}{H_{R}-H_{12}}e^{2jk\left(s+l\right)}$$

where $H_{l}=e^{-jks}$, $H_{R}=e^{jks}$, $j=\sqrt{-1}$, k is the wave number, and s is the distance between the two microphones, l is the distance between test sample and the microphone nearby the sample.

The sound absorption coefficient α and surface acoustic impedance $Z_{s}$ are calculated using the following equations, respectively:

(4) $$\alpha =1-{\left|r\right|}^{2}$$

(5) $$Z_{s}=\rho_{0}c_{0}\frac{1+r}{1-r}$$

where $\rho_{0}$ is the density of air and $c_{0}$ is the speed of sound in air.

The sound absorption coefficient serves as a critical parameter for evaluating the acoustic performance of materials, with values ranging between 0 and 1, where higher values indicate better sound absorption performance [46,47]. For practical engineering applications and rapid comparative assessment, the NRC is commonly employed to provide a comprehensive evaluation of a material’s sound absorption capability. The NRC parameter $\beta_{\mathrm{NRC}}$ is defined as follows [48]:

(6) $$\beta_{\mathrm{NRC}}=\frac{\alpha_{250}+\alpha_{500}+\alpha_{1000}+\alpha_{2000}}{4}$$

where $\alpha_{250}$, $\alpha_{500}$, $\alpha_{1000}$, and $\alpha_{2000}$ are the sound absorption coefficients at the characteristic frequencies of 250 Hz, 500 Hz, 1000 Hz and 2000 Hz, respectively.

#### 2.5.2. Testing Procedure

The testing procedure commenced with system initialization. The geometrical parameters of the impedance tube, including its internal cross-sectional dimensions and the precise distance between the two microphones, were entered into the control software. Based on these parameters, the system automatically determined the compliant test frequency range to ensure effective excitation of one-dimensional plane waves by the acoustic signal.

Subsequently, the output signal was configured, typically selecting a logarithmic chirp signal or white noise covering the determined frequency range as the acoustic excitation source. This was followed by the critical system calibration phase, which consisted of two steps. First, channel calibration was performed to correct for gain deviations in the electronic channels. Then, a precise transfer function calibration was conducted, either by switching the microphones or using a reference sound source, to accurately measure and compensate for amplitude and phase mismatches between the two microphones. This step is crucial for ensuring the accuracy of subsequent wave decomposition.

Following calibration, the test sample was properly installed in the sample holder at the end of the impedance tube, ensuring its front surface was vertical to the tube axis and that peripheral sealing was effective. The test program was then initiated. The system drove the loudspeaker to emit the excitation signal while synchronously acquiring sound pressure data from the two microphones. By calculating the transfer function between them, the incident and reflected waves were separated in real-time, enabling the computation of the material’s normal incidence sound absorption coefficient and acoustic impedance.

To maximize data reliability and repeatability, each sample underwent three consecutive tests under identical conditions. The final reported results represent the average of these three measurements, thereby minimizing random errors.

## 3. Results

### 3.1. Microstructural Analysis

#### 3.1.1. SEM Micro-Morphology

This study successfully fabricated cement-based materials with bio-inspired lamellar structures using the bidirectional ice-templating method. The microstructure of samples, both without admixtures and with a combination of 10% SF and 10%, was comparatively analyzed through scanning electron microscopy (SEM) observations.

**Figure 4:**
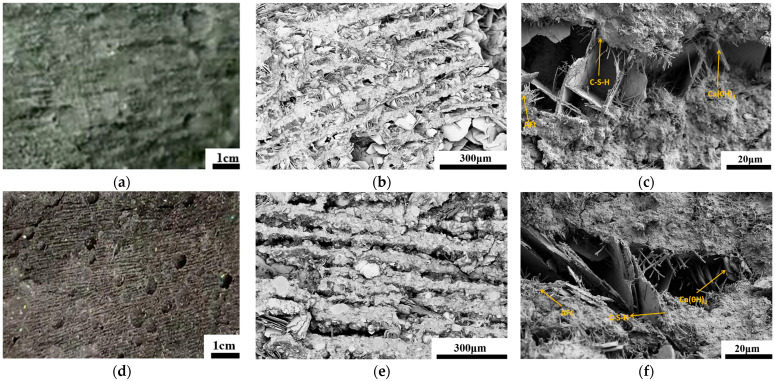
Macroscopic and microstructure of ice-templated cement. (**a**) Macroscopic view of the neat sample. (**b**,**c**) SEM micrographs of the neat sample. (**d**) Macroscopic view of the blended sample (10% SF + 10% FA). (**e**,**f**) SEM micrographs of the blended sample.

In the absence of supplementary materials, SEM images revealed a typical lamellar architecture where cement paste was arranged into parallel platelets separated by aligned, directional pores (Figure 4). These pores originated from the directional growth of ice crystals during freezing and were retained as open channels after thawing. The lamellae surfaces exhibited hydration products including hexagonal portlandite (CH) crystals and needle-like ettringite (AFt). These formed bridges between adjacent lamellae, enhancing the structural integrity. However, some micro-pores remained within the lamellae, partly attributed to water removal during the drying process.

In the sample incorporating 10% SF and 10% FA (at a water-to-binder ratio of 0.8), SEM images displayed a denser and more uniform lamellar structure (Figure 4d–f). The combined action of the micro-filling effect from SF and the morphological effect from FA contributed to a reduction in the size and number of pores within the lamellae. Simultaneously, the spherical particles of FA filled the gaps between cement particles, improving the initial packing density of the paste, while SF participated in pozzolanic reactions, generating additional C-S-H gel that further densified the lamellar structure. Moreover, the incorporation of these admixtures promoted the formation of finer bridging structures between the lamellae, enhancing the interlamellar connectivity.

#### 3.1.2. Pore Structure Analysis

**Figure 5:**
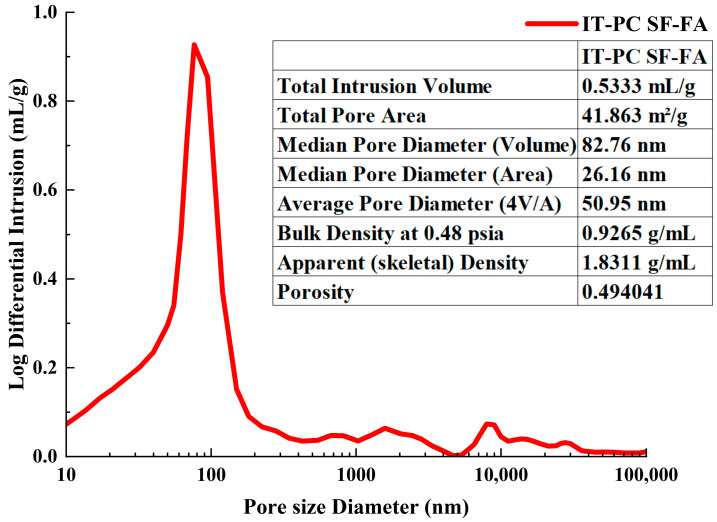
Pore size distribution of the ice-templated cement composite with silica fume and fly ash.

This study quantitatively characterized the pore structure of the ice-templated cement-based composite incorporating SF and FA (IT-PC SF-FA) using Mercury Intrusion Porosimetry (MIP). Key parameters (as shown in Figure 5) include: total pore volume 0.5333 mL/g, total pore area 41.863 m^2^/g, median pore diameter (by volume) 82.76 nm, average pore diameter 50.95 nm, bulk density 0.9265 g/mL, apparent density 1.8311 g/mL, and porosity 49.40%. It should be noted that the pore size diameter in MIP analysis is derived from the volume distribution of intruded mercury. Accordingly, the “median pore diameter (volume)” in the table corresponds to the diameter at which 50% of the total pore volume has been intruded, while the “median pore diameter (area)” is calculated from the cumulative pore surface area distribution. The “average pore diameter (4V/A)” is calculated as four times the total pore volume divided by the total pore area (4V/A), assuming a cylindrical pore model, providing a mean hydraulic size of the pores. These data reveal a typical multi-scale hierarchical pore system, whose structural features can be categorized into three types that collectively determine the material’s macroscopic properties.

The first category is the highly interconnected macro-pore network between cement lamellae, dominantly formed by the ice-templating process. The extremely high permeability (8449 mDarcy) and very low threshold pressure (0.49 psia) provide quantitative evidence for the connectivity of this network, which is also supported by macroscopic and microscopic observations. This aligned open structure provides effective channels for sound wave propagation and stress transfer.

The second category comprises refined mesopores and micropores within the lamellae. Under the synergistic action of SF and FA, the pozzolanic reaction and micro-filling effect led to a denser hydration product structure, further refining the gel pores and concentrating them toward the nanoscale (median pore diameter ~82.76 nm). The MIP data show a sharp peak in pore size distribution centered around 50–100 nm, confirming the dominance of this pore type. In addition, there exists a small number of micrometer-sized pores (tens to hundreds of micrometers) penetrating the lamellae, among which larger ones may disrupt structural continuity.

The third category consists of large-sized closed pores (typically >100 μm) that span multiple lamellae, introduced during the processing. These defects are surrounded by disordered lamellar arrangements and can significantly deteriorate the microstructure and overall performance of the material.

In summary, the material exhibits a unique hierarchical pore structure characterized by “macro-connected yet micro/nano-dense” features. The performance enhancement mechanism lies in the fact that the interconnected macro-skeleton ensures long-range transport and overall dissipation of sound waves/stresses, while the hybrid admixture-induced meso- and micropores provide a greatly increased specific surface area (41.863 m^2^/g), substantially enhancing local viscous energy dissipation (acoustics) and stress dispersion (mechanics). This multi-scale structural design achieved through the synergy of processing and composition regulation is key to breaking through the performance limitations of traditional cement-based materials.

### 3.2. Mechanical Performance Analysis

To systematically evaluate the effects of the ice-templating process and its synergistic interaction with mineral admixtures on the mechanical properties of cement-based materials, this study conducted a comprehensive comparison of samples fabricated by conventional casting (Ref-PC), ice-templating (IT-PC), and ice-templating combined with a blended system of 10% SF and 10% FA (IT-PC SF-FA). Testing was conducted along two critical structural orientations: vertical (V) and parallel (P) to the ice-templated lamellar structure, to reveal the material’s anisotropic characteristics. Through analysis of the flexural and compressive stress-strain curves of each group, the results clearly demonstrate the dual role of the ice-templating method in directionally tailoring material performance and introducing significant anisotropy, while also clarifying the specific contribution of the SF and FA combination within the ice-templated system.

#### 3.2.1. Flexural Performance

**Figure 6:**
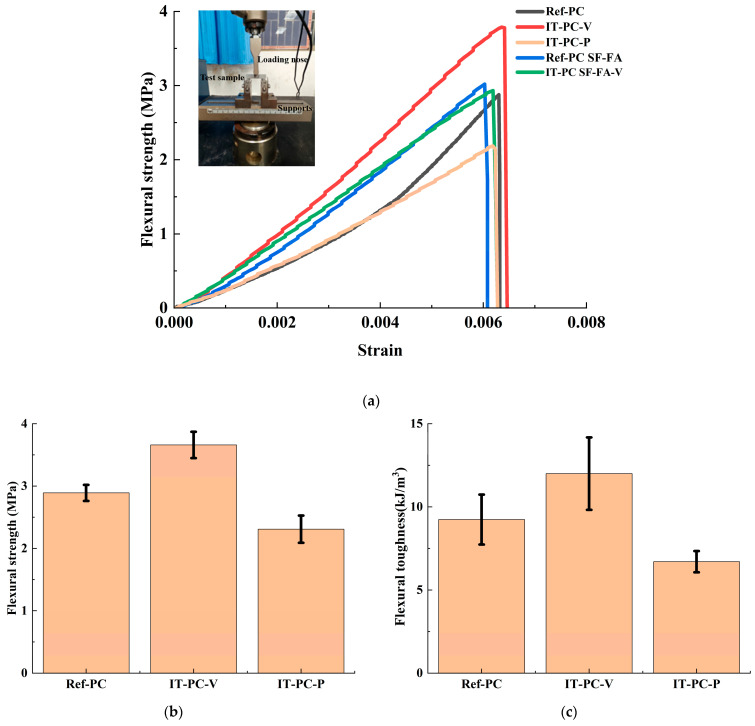

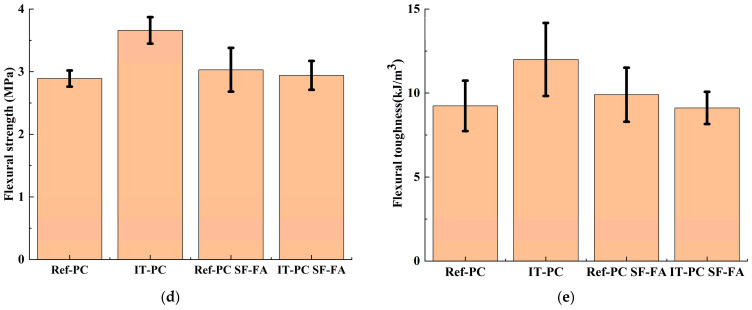
Flexural performance of cement-based materials. (**a**) Flexural stress-strain curves of samples. (**b**) Corresponding flexural strength of the plain samples. (**c**) Corresponding toughness of the plain samples. (**d**) Corresponding flexural strength of the composite samples. (**e**) Corresponding toughness of the composite samples. The data from at least three measurements for each set of samples and presented in form of mean ± standard deviation.

Analysis of the flexural performance reveals that the ice-templating process effectively enhances strength in the vertical direction while introducing pronounced mechanical anisotropy. As shown in Figure 6a,b, the flexural strength of the IT-PC-V sample—loaded vertical to the lamellar structure—is significantly higher than that of the conventionally cast Ref-PC and the parallel-loaded IT-PC-P sample. Quantitatively, the Ref-PC sample exhibited a flexural strength of 2.89 ± 0.13 MPa and a toughness of 9.23 ± 1.5 kJ/m^3^, serving as the benchmark. In contrast, the IT-PC-V sample achieved a markedly higher flexural strength of 3.66 ± 0.21 MPa and toughness of 12 ± 2.18 kJ/m^3^, while the IT-PC-P sample showed lower values of 2.31 ± 0.22 MPa and 6.7 ± 0.64 kJ/m^3^, respectively. This directional enhancement is attributed to the failure mechanism governed by the aligned porous architecture: under vertical loading (IT-PC-V), crack propagation is forced to traverse and fracture the continuously bridged pore walls, dissipating considerable energy and thereby improving both strength and toughness. In contrast, under horizontal loading (IT-PC-P), cracks readily propagate along the weak lamellar interfaces, leading to stress concentration and brittle failure with lower strength. The Ref-PC sample exhibited typical brittle behavior of a homogeneous material, with intermediate strength between the two directional samples. These results confirm that ice-templating enables performance customization along specific loading directions, achieving approximately 26.6% higher flexural strength and 30% greater toughness in the direction vertical to the lamellae compared to conventional cement.

A key conclusion drawn from Figure 6a,d is that the incorporation of SF and FA does not further enhance the absolute flexural strength in the optimal orientation (IT-PC SF-FA-V) within the ice-templated lamellar structure. Specifically, the IT-PC-V sample exhibited the highest flexural strength among all groups, slightly surpassing that of IT-PC SF-FA-V. This is quantitatively supported by the measured flexural strength and toughness of IT-PC SF-FA-V (2.94 ± 0.23 MPa and 9.11 ± 0.96 kJ/m^3^, respectively), which are lower than those of IT-PC-V. For reference, the conventionally cast composite (Ref-PC SF-FA) exhibited values of 3.03 ± 0.36 MPa and 9.9 ± 1.61 kJ/m^3^.This observation aligns with previous findings that SF offers limited improvement to the overall strength of ice-templated cement [26]. The underlying reason lies in the dominant role of the macro-scale interlaminar pore structure in determining mechanical properties, as captured by the “equivalent element model,” where strength is primarily governed by total and interlaminar porosity. Although SF and FA densify the microstructure of cement lamellae through micro-aggregate filling and pozzolanic reactions (as shown in the SEM images in Section 3.1), such intra-lamellar strengthening contributes marginally compared to the decisive influence of the macroscopic lamellar architecture. Consequently, the strength of IT-PC SF-FA-V does not exceed that of IT-PC-V.

In summary, the ice-templating method plays the central role in conferring excellent flexural performance, particularly in the direction vertical to the lamellae. In the system studied here, the incorporation of 10% SF and 10% FA contribute primarily to environmental benefits rather than mechanical enhancement, offering a new approach to designing eco-friendly, lightweight cement-based materials with tailored performance.

#### 3.2.2. Compressive Performance

**Figure 7:**
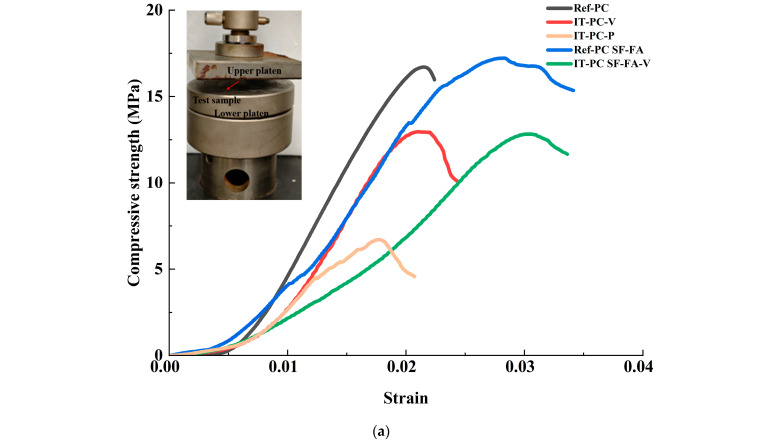

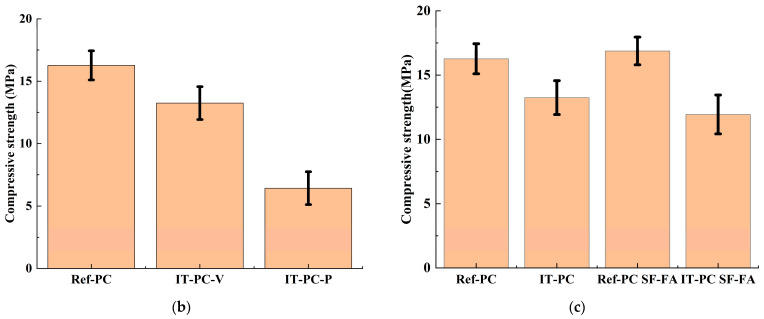
Compressive performance of cement-based materials. (**a**) Compressive stress-strain curves of samples. (**b**) Corresponding compressive strength of the plain samples. (**c**) Corresponding compressive strength of the composite samples. The data from at least three measurements for each set of samples and presented in form of mean ± standard deviation.

Analysis of the compressive behavior reveals that although the ice-templating method introduces significant anisotropy, the compressive strength is lower in all loading directions compared to conventionally cast samples. As shown in Figure 7a,b, both the IT-PC-V sample (loaded vertically to the lamellar structure) and the IT-PC-P sample (loaded parallel to the lamellar structure) exhibit significantly lower compressive strength than the dense Ref-PC sample. This is quantitatively confirmed by the measured compressive strengths: Ref-PC at 16.00 ± 0.13 MPa, IT-PC-V at 13.25 ± 1.32 MPa, and IT-PC-P at 6.43 ± 1.31 MPa. Notably, the incorporation of SF and FA positively influences the deformation characteristics of the material. In the conventional casting process, the Ref-PC SF-FA sample demonstrates a higher peak strain compared to Ref-PC. Similarly, within the ice-templated system, the IT-PC SF-FA-V sample also shows an increase in peak strain relative to IT-PC-V. This indicates that the composite admixture strategy effectively enhances the deformability of the material under different processing conditions. However, as shown in Figure 7c, the effectiveness of the composite admixture strategy in improving strength within the ice-templated system is limited. The compressive strength of IT-PC SF-FA-V does not show significant improvement compared to IT-PC-V. This is substantiated by the measured compressive strengths of 16.88 ± 1.08 MPa for Ref-PC SF-FA and 11.94 ± 1.51 MPa for IT-PC SF-FA-V. This observation aligns with findings in the literature [26], which report that SF provides limited enhancement to the overall strength of ice-templated cement. The underlying mechanism lies in the dominant role of the macro-porous structure in controlling compressive failure. Although the admixtures densify the lamellar matrix through micro-filling and pozzolanic reactions (as evidenced by SEM results in Section 3.1) and increase the peak strain under both processing methods, this micro-scale strengthening is insufficient to compensate for the failure mode dictated by the macro-porous architecture in the ice-templated system.

In summary, ice-templated cement exhibits unique anisotropic behavior under compressive loading but demonstrates lower strength in all directions compared to conventionally cast samples. The strategy of incorporating SF and FA consistently improves the peak strain—and thus the deformability—in both conventional and ice-templated systems, yet it fails to effectively enhance the compressive strength within the ice-templated system. This further confirms the predominant role of the macroscopic structure in determining the compressive performance of ice-templated systems. Compared to the “wood-inspired cement” prepared by Wang et al. [26] using ice-templating with 10% SF addition, this study, under similar ice-templating conditions, achieves a comparable trend in mechanical enhancement while further endowing the material with excellent anisotropic bending properties and outstanding sound absorption functionality through the hybrid incorporation of 10% SF and 10% FA. This not only validates the broad applicability of the ice-templating method in regulating mechanical performance but also expands the functional scope of the material by introducing composite admixtures. In terms of structural design and performance enhancement, Chen et al. separately fabricated cement-hydrogel [24] and cement-epoxy multilayer composites [27] using bidirectional ice-templating, achieving significant improvements in toughness and strength through vacuum-assisted impregnation. While this study does not introduce polymer impregnation, the hybrid incorporation of 20% industrial solid waste maintains a similar mechanical enhancement trend while significantly improving the material’s environmental friendliness and cost-effectiveness, offering new insights for the development of green building materials. Compared to existing research, this work further extends the functional dimensions and environmental benefits of ice-templated cement-based materials.

### 3.3. Sound Absorption Performance Analysis

To systematically investigate the synergistic effects of the ice-templating method and mineral admixtures (SF, and FA on the acoustic properties of cement-based materials, this study comparatively analyzed the sound absorption coefficient versus frequency curves and NRC of samples with different processing methods and formulations using an impedance tube testing system.

#### 3.3.1. Acoustic Effects of the Anisotropic Ice-Templated Structure

**Figure 8:**
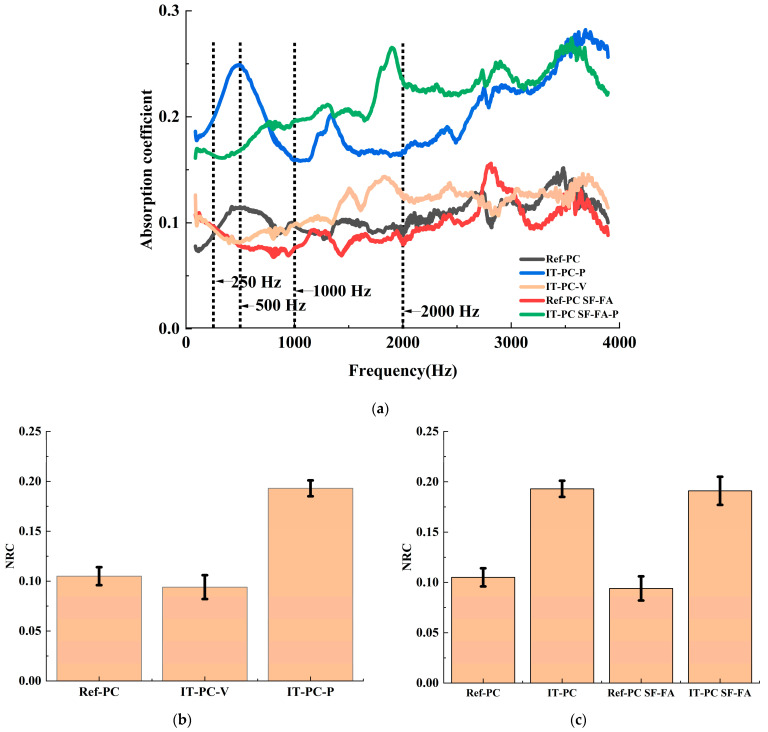
Sound absorption performance of the cement-based materials. (**a**) Sound absorption coefficient versus frequency curves of samples (**b**) Corresponding Noise Reduction Coefficient (NRC) values of the plain samples. (**c**) Corresponding Noise Reduction Coefficient (NRC) values of the composite samples. The data from at least three measurements for each set of samples and presented in form of mean ± standard deviation.

This section systematically evaluates the impact of the ice-templating process and its introduced anisotropic structure on the sound absorption performance of porous cement-based materials using the impedance tube testing system. Figure 8a displays the variation of the sound absorption coefficient with frequency for different samples. Overall, the sound absorption coefficient of all samples increases with rising frequency, exhibiting the typical sound absorption characteristics of porous materials. However, significant performance differences exist among samples with different processes and orientations. Firstly, the reference sample prepared by ice-templating (IT-PC) demonstrates markedly higher sound absorption coefficients across all frequency bands compared to the conventionally cast reference sample (Ref-PC). This directly confirms the superior efficacy of the unique directional porous structure created by ice-templating method in dissipating sound energy. More importantly, the ice-templated sample exhibits distinctly different sound absorption behaviors when tested along different orientations: the sound absorption performance along the ice growth direction (IT-PC-P) is significantly superior to that in the vertical direction (IT-PC-V). This pronounced anisotropy proves that sound energy dissipates more readily when acoustic waves propagate parallel to the aligned pore channels, establishing the structural foundation for the targeted optimization of acoustic performance via ice-templating. To facilitate a more direct comparison of the overall sound absorption performance, Figure 8b presents the NRC for each sample. The measured NRC values were 0.105 ± 0.009 for Ref-PC, 0.09 ± 0.009 for IT-PC-V, and 0.193 ± 0.008 for IT-PC-P. The NRC value of the ice-templated reference sample (IT-PC) substantially exceeds that of the conventional sample (Ref-PC). Specifically, the NRC value for IT-PC-P is approximately 94% higher than that of Ref-PC and about 114% higher than that of IT-PC-V. This provides definitive numerical evidence that ice-templating method significantly enhances sound absorption performance and introduces pronounced acoustic anisotropy.

#### 3.3.2. Synergistic Regulation Mechanism of Silica Fume and Fly Ash

To investigate the regulatory mechanism of mineral admixtures (SF and FA) and their synergy with the preparation process on acoustic performance, this study further compared and analyzed the sound absorption performance of the composite admixture strategy under different process systems. The results indicate that the acoustic effect of the composite admixture strategy strongly depends on the macro-fabrication process of the material system. In the conventional casting system (Ref-PC), the incorporation of SF and FA inhibits sound absorption performance. As shown in Figure 8c, the NRC of Ref-PC SF-FA is lower than that of the reference group Ref-PC, with a measured value of 0.094 ± 0.012. This phenomenon can be attributed to the “densification effect” of SF and FA in the traditional cement system. The ultra-fine particles of SF and the spherical particles of FA effectively block and refine the capillary pores within the paste, significantly reducing pore connectivity and pore size, making it more difficult for sound waves to penetrate the material interior for energy dissipation, leading to decreased sound absorption performance. In contrast, within the ice-templated (IT-PC) system, the combination of SF and FA forms a “golden combination” for achieving high-performance sound absorption. As shown in Figure 8a,c, the NRC value of IT-PC SF-FA approaches the highest level among all groups, with a value of 0.191 ± 0.014, demonstrates an 82% improvement compared to Ref-PC SF-FA, and exhibits superior broadband sound absorption characteristics. 

The internal structure of the ice-templated sample with the hybrid blend features a macroscopically interconnected lamellar framework, which provides efficient channels for sound wave propagation and energy transmission. Simultaneously, the incorporation of the blend creates a dense network of micro-scale pores within this lamellar structure, increasing the paths for sound wave reflection and scattering, as well as the surface area for frictional dissipation. This enhances the overall sound absorption performance and improves the NRC. In contrast, the internal pores of the sample prepared by the conventional process are very fine. The addition of the hybrid blend under these conditions leads to an even denser microstructure, which impedes sound waves from penetrating the sample, thereby preventing effective absorption and resulting in reduced sound absorption performance. This exceptional performance stems from the hierarchical “macro-micro” pore structure co-constructed by the unique forming process of ice-templating and the synergistic action of SF/FA. The ice-templating method constructs a highly interconnected macro-porous skeleton, while the addition of admixtures, on one hand, increases the slurry viscosity [49], inhibiting ice crystal growth and refining the macro-channels, thereby enhancing the viscous dissipation of mid-to-high frequency sound waves. On the other hand, pozzolanic reactions generate a multitude of micro/nano-scale secondary pores within the pore walls (Figure 4). This hierarchical pore structure, characterized by “macro-scale connectivity and micro-scale distribution,” synergistically enables highly efficient broadband sound absorption from low to high frequencies. This analysis reveals a core mechanism: the impact of SF and FA on the acoustic performance of cement-based materials is not determined by their intrinsic properties alone but is strongly dependent on the macro-fabrication process of the material system. This finding clarifies that the combination of “macro-structural construction via ice-templating and micro-structural optimization via mineral admixtures” presents a highly promising strategy for fabricating high-performance, broadband cement-based sound-absorbing materials.

Regarding sound-absorbing materials, many existing studies face the common challenge of balancing sound absorption performance with mechanical strength. Wang et al. prepared porous sound-absorbing cement using calcium alginate hydrogel as a pore-forming agent [50]. Although their material exhibited excellent acoustic performance, its compressive strength was only 1.31 MPa, severely limiting its potential for structural applications. Liang et al. studied cement-based ceramsite sound-absorbing materials [51], reporting a compressive strength of 11.47 MPa, which remains lower than that of our ice-templated composite material. Furthermore, our material possesses the unique advantage of tunable anisotropy, which is crucial for applications with specific requirements for directional load-bearing or sound wave propagation.

## 4. Conclusions

This study employs the ice-templating method to fabricate novel cement-based materials incorporating silica fume and fly ash. The effects of the fabrication process and the fly ash/silica fume hybrid blend on the meso-structure, mechanical properties, and sound absorption performance of the materials were systematically investigated. The main conclusions are as follows:

(1) The ice-templating process successfully constructed a bio-inspired layered structure within the fly ash- and silica fume-incorporated cement matrix, imparting significant mechanical anisotropy to the material. In the direction vertical to the lamellae, the flexural performance was remarkably enhanced, with the flexural strength and toughness increasing by approximately 26.6% and 30%, respectively. This improvement is attributed to the fact that crack propagation should traverse continuous pore walls, thereby dissipating more energy. However, this porous structure also reduced the compressive strength. The ice-templated method is effective to tailor performances of materials. Consequently, this process is highly suitable for applications involving well-defined unidirectional loads.
(2) The influence of the fly ash/silica fume hybrid blend on the mechanical properties and environmental benefits of the ice-templated cement materials presents a dual effect. While the overall strength trend is predominantly governed by the internal structure regulated by the ice-templating method, the hybrid blend increases the compressive peak strain of the cement material. This indicates an effective enhancement of the material’s deformability and an improvement in the brittleness typical of cement-based materials. More importantly, this strategy utilizes a combination of 10% fly ash and 10% silica fume to replace cement equivalently with industrial solid waste, significantly reducing the material’s carbon footprint. It thereby provides a practical and viable pathway towards the green, economical, and high-value-added resource utilization of building materials.
(3) The cement materials incorporating fly ash and silica fume fabricated via the ice-templating method exhibit a significant improvement in sound absorption performance. Acoustic impedance tube tests demonstrated that the NRC of the novel cement-based material increased by 82%. This enhancement is attributed to the synergistic effect of the ice-templating method and the hybrid blend: the aligned macro-pores created by ice-templating method provide efficient channels for sound wave propagation and dissipation. Simultaneously, the hybrid blend, by increasing paste viscosity and through pozzolanic reactions, synergistically refines the macro-channels and introduces micro/nano-scale secondary porosity. Together, they form an ideal hierarchical sound-absorbing structure characterized by “macroscopically interconnected and microscopically dense” pores. This demonstrates that a strategy combining “macro-structural design” with “micro-component optimization” can overcome the traditional trade-off between mechanical and functional properties in materials, establishing a new paradigm for developing high-performance building acoustic materials.
(4) Practical application perspective: The current work still has certain limitations, including the lack of systematic quantitative control over key process parameters (such as freezing rate and temperature gradient) during ice-templating, the unassessed long-term durability of the material under complex environmental conditions, and the yet-to-be-established acoustic theoretical modeling based on the multi-scale pore structure. Owing to its tunable anisotropy, well-balanced overall performance, and significant solid waste utilization benefits (with a total substitution rate of 20%), the material demonstrates clear application potential in architectural acoustics and green building fields, such as in lightweight sound-absorbing wall panels, traffic noise barriers, and building envelope systems.

## Figures and Tables

**Table 1:** Chemical compositions of raw materials.

Raw Material	Mass Fraction/wt%									
	CaO	SiO_2_	Al_2_O_3_	Fe_2_O_3_	SO_3_	K_2_O	MgO	LOI	Free CaO	Other
Cement	56.72	22.68	7.36	3.98	3.94	1.48	1.09	2.14	-	0.61
Silica fume	-	98.10	-	-	-	-	-	1.48	-	0.42
Fly ash	5.60	43.00	24.00	2.50	0.80	-	0.93	3.20	0.80	19.17

## Data Availability

The original contributions presented in this study are included in the article. Further inquiries can be directed to the corresponding author.
